# A Case of False-Positive HIV Test in a Patient With Newly Diagnosed Hodgkin Lymphoma and Literature Review

**DOI:** 10.7759/cureus.10884

**Published:** 2020-10-10

**Authors:** Sumera Bukhari, Ahmed Dirweesh, Afolarin Amodu, Muhammad Nadeem, Sara L Wallach

**Affiliations:** 1 Internal Medicine/Hospital Medicine/Palliative Medicine, Cambridge Health Alliance/Harvard Medical School, Cambridge, USA; 2 Division of Gastroenterology, Hepatology and Nutrition, University of Minnesota, Minneapolis, USA; 3 Internal Medicine: Nephrology, Boston University Medical Center, Boston, USA; 4 Internal Medicine, Seton Hall University, Saint Francis Medical Center, Trenton, USA

**Keywords:** hiv testing, false hiv test, hodgkin lymphoma, mediastinal mass

## Abstract

Hodgkin lymphoma (HL) is one of the non-acquired immunodeficiency syndrome (AIDS)-defining cancers (NADCs). HIV testing has become a part of routine testing in HL because of commonly anticipated association. Here we report an unusual case where the need for HIV screening in a newly diagnosed case of HL raised an ethical dilemma and a medical challenge due to false-positive HIV test results. In literature, pregnancy, autoimmune disorders, some viral infections, and the presence of hypergammopathy of hematologic malignancy have all been linked with false-positive HIV screening. The reactive results require additional testing with an HIV-1/HIV-2 antibody differentiation assay. The specimens show reactivity on the initial screening immunoassay, but negative or indeterminate antibody differentiation assay should undergo nucleic acid testing. Nevertheless, several instances of discordance between screening and confirmatory techniques have been described. It is speculated that this might be due to coincidental cross-reaction of subtypes of polyclonal gamma globulin with the HIV p24 antigen. In conclusion, this case signifies the understanding of the HIV testing algorithm and the use of reflex testing in the context of a positive HIV test before disclosing such preliminary results to patients and/or physicians.

## Introduction

The HIV-infected individuals have an increased predilection for growing malignancy [1]. The United States Centers for Disease Control (CDC) has determined Kaposi sarcoma, non-Hodgkin lymphoma, and cervical cancer as the "acquired immunodeficiency syndrome (AIDS) defining cancers." A diagnosis of any one of these cancers marks the point at which HIV infection has progressed to AIDS. Also, people infected with HIV are at higher risk of several other types of cancer than the general population, and these are collectively described as non-AIDS-defining cancers (NADCs). These other malignancies include anal, liver, lung cancer, and Hodgkin lymphoma (HL) [2]. Thus, HIV testing is a part of routine testing in HL because of commonly anticipated association. Here, we report a compelling case of a patient with recently diagnosed HL, who found to be falsely positive for HIV on an initial diagnostic workup for HL.

## Case presentation

A 38-year-old male with no significant past medical history presented to the ER with the chief complaint of a five-month history of hemoptysis. The hemoptysis is associated with mild right-sided chest pain, fever, night sweats, and unintentional weight loss (15 pounds in the last three months). He denied shortness of breath, sick contacts, and travel or incarceration history. He had no history of tobacco, drug, or alcohol use. He was in a monogamous relationship, and his family history was noncontributory.

On examination, vitals were normal with a blood pressure of 112/60 mmHg, pulse rate of 80 beats/min, respiratory rate of 16 breaths/min, temperature of 98.5 degree Farenheit, and BMI of 19.1 with no palpable lymphadenopathy. Dull percussion was noted in the right middle lung zones. The rest of the clinical examination was normal. His blood workup showed a hemoglobin of 11.3 g/dL, white blood cells (WBCs) of 7200/mm3, and platelet count of 337 x 103/mm3. His lactate dehydrogenase (LDH) was normal at 161; his blood urea nitrogen (BUN), creatinine, and liver function tests were within normal ranges. Epstein-Barr virus (EBV) titers were negative.

**Figure 1:**
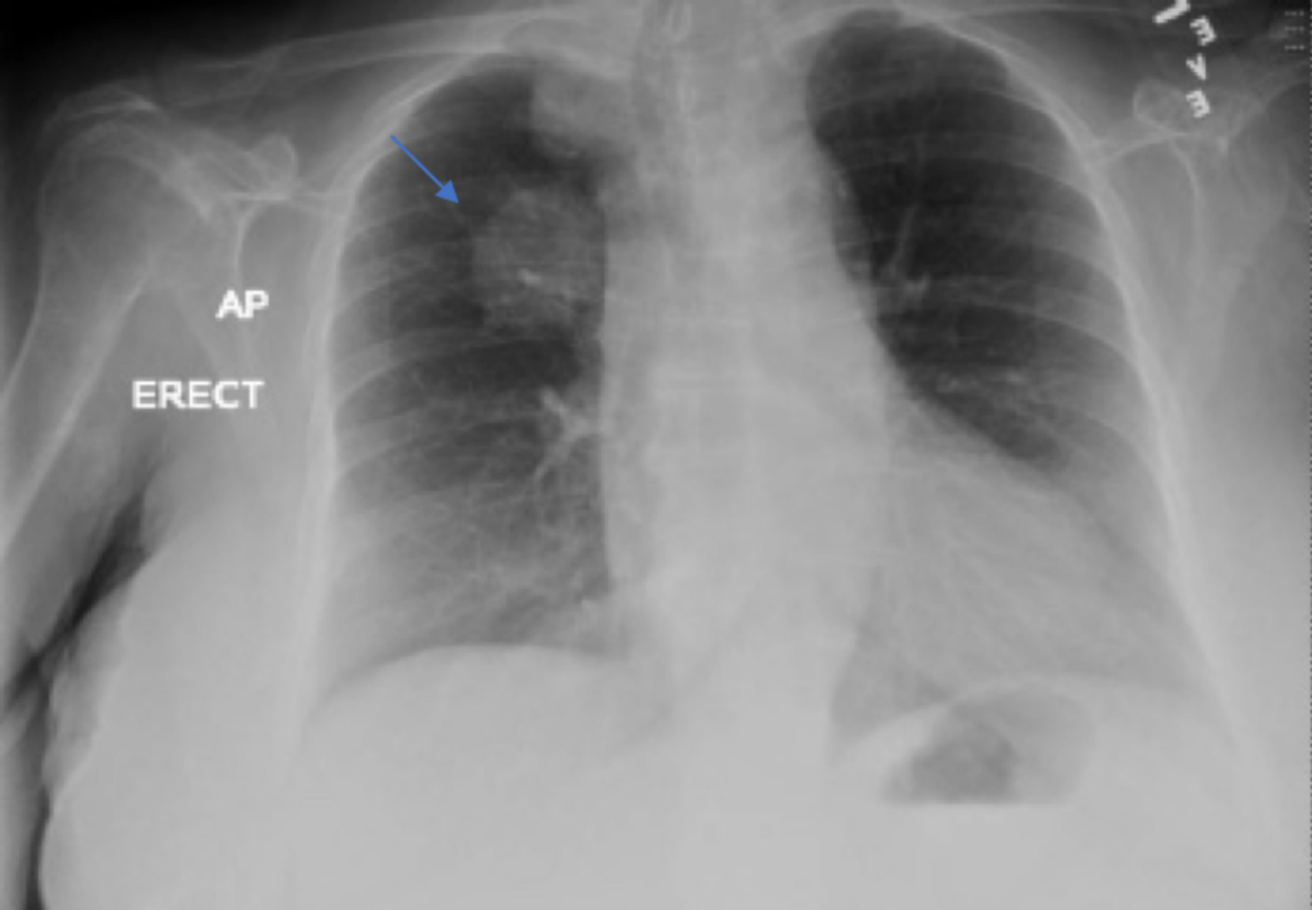
Chest X-ray.

Chest X-ray (CXR) showed 8.8 cm x 5.9 cm lobulated right para hilar/right para-cardiac mass consistent with malignancy (Figure 1).

**Figure 2:**
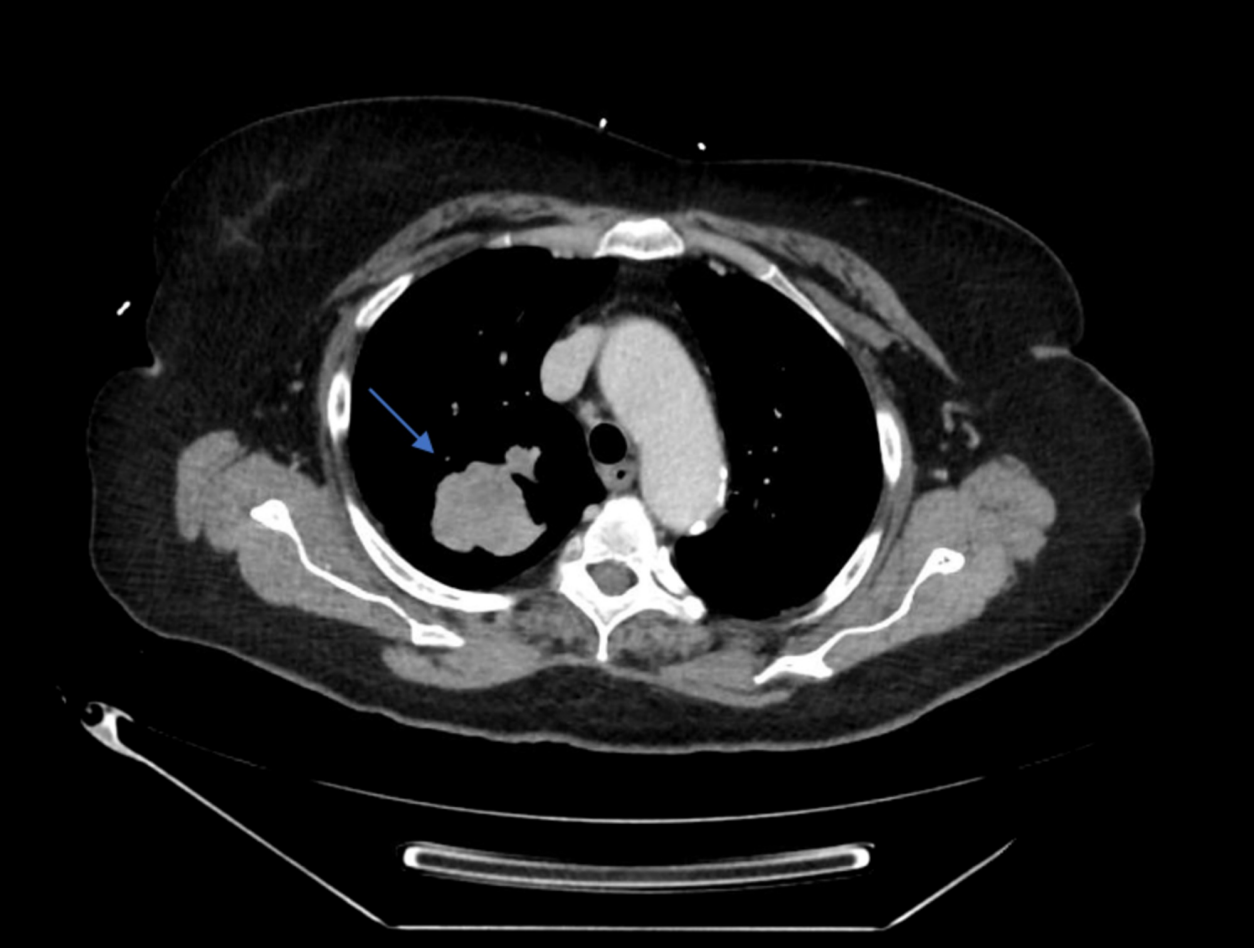
CT scan of chest.

The CT image of the chest revealed a large right upper lobe mass encasing the bronchi and major vessels in the right lower lobe and right middle lobe (Figure 2).

There were also multiple right lung nodules, predominantly pleural-based, likely representing lymphomatous involvement within the lung. A mediastinoscopy with biopsy showed HL (mixed cellularity type) with positive CD30 and CD15, weakly positive PAX-5, and a negative leukocyte common antigen (LCA). There was no phenotypic evidence of blasts, myeloid dysmaturation, or non-Hodgkin lymphoma, and stains for acid-fast bacilli and fungus were negative. CT scan of the abdomen showed mesenteric and retroperitoneal lymphadenopathy with splenic lesions. A patient-confirmed HIV-1 and HIV-2 antibodies with P24 antigen were reactive, with an undetectable viral load. A sequential repeat antigen-antibody test before the start of chemotherapy came back negative. 

## Discussion

Hodgkin lymphoma is one of the NADCs, and its incidence with HIV infection may have increased as HIV-infected patients are living longer and also substantially with the use of combination antiretroviral therapy (cART) [2]. Thus, HIV testing is a part of routine testing in HL because of this commonly anticipated association. Interestingly, the pattern of histologic subtypes of HL seen in HIV-infected patients has a higher proportion of lymphocyte depletion (LD) and mixed cellularity (MC) [3-4]. Both subtypes of classical HL are related to more advanced immune compromise, while nodular sclerosis (NS) histology increases with higher CD4 counts than cART [2]. It becomes crucial to test for HIV after diagnosing lymphoma, as the disease is more aggressive in HIV patients, and response to chemotherapy can be affected by HIV due to certain factors. Levine et al. [5] studied the decreased response rates and inferior outcomes during the pre-cART era due to dose reductions and delays, higher-risk disease, and increased treatment-related morbidity mortality. The administration of chemotherapy with cART proved feasible. It exhibited an increased ability to cure HL in a subset of HIV-infected patients in a study by Errante et al. [6]. 

Burke et al. [7] studied the frequency of false-positive diagnoses retrospectively among applicants seropositive for HIV in a subpopulation with a very low infection prevalence. They concluded that screening programs for HIV infection in a population with a known low prevalence of infection could have an acceptably low false-positive rate. The HIV enzyme-linked immunosorbent assay (ELISA) test is a sensitive test used for screening purposes; however, heterophile antibodies have been linked with many immunoassays' erroneous results [8-9]. Therefore, the ELISA test can be falsely positive; there is a need to use other confirmatory tests. Although there are reports of cross-reaction with gp41 and p24 on Western blots (WBs) in low-risk blood donors [10], it has been estimated that up to 0.5% of patients who are seropositive for HIV turn out to be false positive. In contrast, the WB has been traditionally used for confirmation; a positive WB may represent cross-reactivity with non-HIV antibodies present in HIV patients and those at risk [11]. Multispot antibody differentiation test has been tested to have specificity and sensitivity more significant than 99% [12], comparing satisfactorily with standard Western blotting as a confirmatory assay [13].

Besides, pregnancy, autoimmune disorders, some viral infections, and the presence of hypergammopathy of hematologic malignancy have all been linked with false-positive HIV screening [14-17] and, therefore, should have some form of confirmatory test done. The CDC, in 2014, updated recommendations for the diagnosis of HIV infection to include an innovative algorithm using the fourth-generation screening and confirmatory assays [18]. As per the recommendations, initial screening comprises a combination immunoassay or fourth-generation test that uses the detection of both HIV-1/HIV-2 antibodies along with HIV-1 p24 antigen assay. Negative test results conclude the testing algorithm. However, reactive results require additional testing with an HIV-1/HIV-2 antibody differentiation assay. The specimens show reactivity on the initial screening immunoassay, but negative or indeterminate antibody differentiation assay should undergo nucleic acid testing. It is biologically reasonable that disorders that would yield a false-positive fourth generation antigen/antibody assay could cause a false-positive antibody differentiation assay. Simultaneously, reported sensitivity and specificity of fourth-generation tests remain above 99% in many trials, with several discords between screening and confirmatory techniques described. 

The literature projects several speculations about the mechanism of cross-reactivity believed to be responsible for the false-positive reports. Investigators have speculated that antigenic mimicry between the individual's epitopes and retroviral antigens is the reactivity mechanism for HIV p24 antigen. Muta et al. reported false-positive HIV serology in angioimmunoblastic T-cell lymphoma (AITL) patients and speculated that this could be attributed to a coincidental association of polyclonal gamma globulin subtypes with the HIV p24 antigen [19]. The exact mechanism that is responsible for the false-positive antibody reaction, in this case, is unclear; it may also be related to the HIV p24 antigen cross-reactivity as speculated in other cases described in AITL and autoimmune disorders.

## Conclusions

In conclusion, this case reflects the need for reflex testing after an initial positive HIV test result and emphasizes the need for understanding HIV testing at a molecular level. It stresses the practice of confirmatory tests before disclosing such results to patients, which raises an ethical dilemma and a medical challenge due to false-positive HIV test results.
